# Noise exposure modulates cochlear inner hair cell ribbon volumes, correlating with changes in auditory measures in the FVB/nJ mouse

**DOI:** 10.1038/srep25056

**Published:** 2016-05-10

**Authors:** Stephen T. Paquette, Felicia Gilels, Patricia M. White

**Affiliations:** 1 Department of Neuroscience, University of Rochester School of Medicine and Dentistry, Box 603, 601 Elmwood Avenue, Rochester, NY, 14642, USA

## Abstract

Cochlear neuropathy resulting from unsafe noise exposure is a life altering condition that affects many people. This hearing dysfunction follows a conserved mechanism where inner hair cell synapses are lost, termed cochlear synaptopathy. Here we investigate cochlear synaptopathy in the FVB/nJ mouse strain as a prelude for the investigation of candidate genetic mutations for noise damage susceptibility. We used measurements of auditory brainstem response (ABR) and distortion product otoacoustic emissions (DPOAE) to assess hearing recovery in FVB/nJ mice exposed to two different noise levels. We also utilized confocal fluorescence microscopy in mapped whole mount cochlear tissue, in conjunction with deconvolution and three-dimensional modeling, to analyze numbers, volumes and positions of paired synaptic components. We find evidence for significant synapse reorganization in response to both synaptopathic and sub-synaptopathic noise exposures in FVB/nJ. Specifically, we find that the modulation in volume of very small synaptic ribbons correlates with the presence of reduced ABR peak one amplitudes in both levels of noise exposures. These experiments define the use of FVB/nJ mice for further genetic investigations into the mechanisms of noise damage. They further suggest that in the cochlea, neuronal-inner hair cell connections may dynamically reshape as part of the noise response.

Hearing dysfunction arises when the auditory system fails to properly convey acoustic signals to the brain. Of the many human conditions which fall under this description, the sensorineural hearing loss otherwise known as auditory neuropathy spectrum disorder, may be the most insidious and difficult to treat. The many different forms of neuropathy observed within the cochlea can be thought of as organized around the particular sensory element causing the dysfunction. Specific points at which signal conveyance may be disrupted include synaptic loss, which is termed cochlear synaptopathy^1^, loss of sensory inner hair cells within the Organ of Corti termed cochlear neuropathy, the loss of sensory neurons in the spiral ganglion, or demyelination of auditory nerve fibers within the cochlear nerve.

Cochlear synaptopathy is one of the pathologies caused by acoustic overexposures. Noise-induced hearing neuropathies have been reported as causing up to 90% of hearing deficits amongst the elderly^2^. This is of note because the most common complaint associated with hearing neuropathy is the inability to understand speech, particularly in noisy environments. This leads to an overall declining quality of life with associated adult learning problems and may hinder medical treatment outcomes due to communication deficits. In addition, noise induced hearing dysfunction has been reported at rates as high as 3 in 5 for active and retired military personnel^3^.

Diagnosis of cochlear synaptopathy as a result of auditory exposure in patients is particularly difficult. Hearing disorders are commonly assessed with auditory brainstem response (ABR) and distortion product otoacoustic emissions (DPOAE) tests, but patients may have normal thresholds for both. Other methods of diagnosis, such as word recognition scoring and speech perception in noise scoring have also been used^4^. Speech perception cannot be assessed in animals; however, a reduced ABR waveform peak 1 without affected DPOAE has been documented in rodents after noise exposure^5^. The latter effects on suprathreshold responses may be used as a proxy to assess the hearing dysfunction that accompanies this common insult for human and rodent. Such a reduction has been directly correlated with a loss of synapses between the inner hair cell and spiral ganglion neurons^1^, suggesting a causal role for synaptic loss in noise overexposure-induced cochlear neuropathy. However, reduced ABR amplitudes may still occur in the absence of synapse loss^6^.

Mouse models remain the predominant method by which to assess the anatomical pathologies of noise damage, and it will be interesting to apply the power of mouse genetics to this issue. Most studies of noise damage have been performed on the CBA/CaJ mouse lineage, which otherwise maintains good hearing into old age^7^. As such, this is the “gold standard” mouse model for hearing research. However, few genetic models are bred to this line. Several congenic mouse strains commonly used as genetic models, such as C57BL/6J and 129/SvEv, are ill-suited for noise damage experiments. C57BL/6J carries the Pchd23 mutation, which sensitizes outer hair cells to damage from noise^8^. 129/SvEv mice exhibit a well-known resistance to noise damage^9^. In contrast, the FVB/nJ mouse line is well suited for noise damage analysis. First, like CBA/CaJ and C57BL/6J, FVB/nJ is congenic^10^ and therefore unique phenotypes resulting from genetic manipulations are not complicated by inherent genetic variation^11^. Second, FVB/nJ hearing thresholds are stable for the first 10 months of life^12^. Third, FVB/nJ mice have a youthful sensitivity to noise damage, similar to CBA/CaJ^13^. Fourth, there are a large number of well-characterized genetic mutations on this mouse strain for technical reasons^14^,^15^,^16^,^17^. In order to identify the molecular pathways that maintain auditory function in the face of insult, it is necessary to characterize a useful genetic lineage in mice. However, the anatomical correlates of noise damage have not been documented for the FVB/nJ line.

Here we correlate changes in hearing measures with changes in pre-and post-synaptic markers in noise-exposed FVB/nJ mice. We use two noise exposure conditions, one that establishes a reduction in peak 1 amplitude without significant synaptic losses as well as one where both sequelae are observed. We analyze synaptic components via three-dimensional reconstructions of deconvolved confocal microscopy stacks. The volumes of pre-synaptic ribbons and post-synaptic receptor patches are each dynamically regulated in both conditions two weeks post-exposure. In particular, ribbons appear to exist in multiple size distributions which are differentially affected in response to each noise condition. The precise positioning of synapses on the inner hair cell also appears affected. These data provide an anatomical correlate for reductions in peak 1 amplitude in the absence of synaptic loss, and define conditions where FVB/nJ mice may be used to understand the genetic basis of noise damage.

## Results

### FVB/nJ mice as a mouse model for sensitivity to acoustic over-exposure

**Figure 1:**
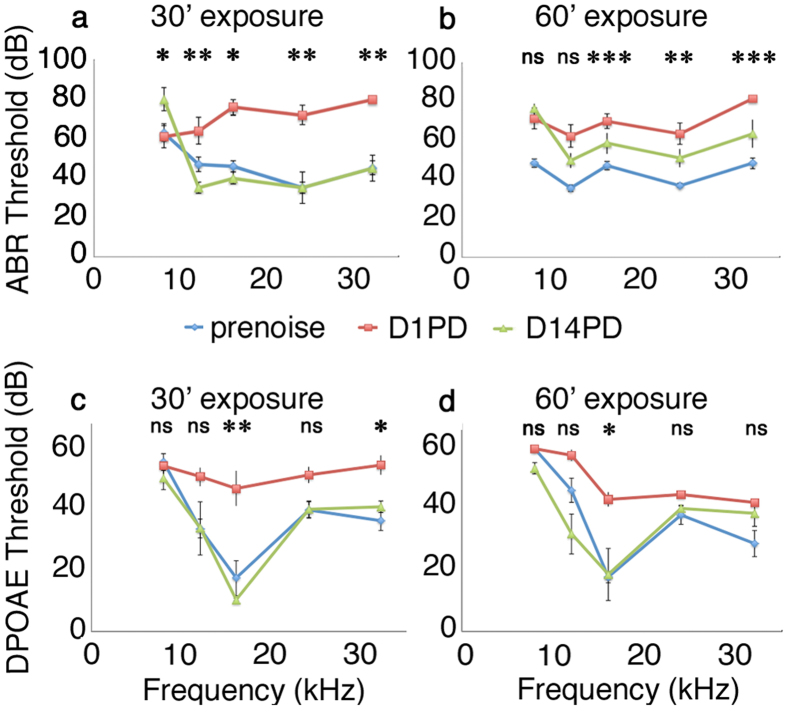
Varied noise exposure conditions produce temporary and permanent threshold shifts in FVB/nJ mice. (**a,b**) ABR thresholds from mice prior (cyan, prenoise), one day after (red, Day 1 post deafening (D1PD) and 14 days after (green, D14PD) a (**a**) 30 minute noise exposure or a (**b**) 60 minute noise exposure (**b**). Thresholds rise one day after noise exposure, and recover either fully or partially fourteen days later. (**c,d**) DPOAE thresholds from the same mice shown in (**a,b**). DPOAE thresholds rise one day after noise exposure, and recover completely 14 days later. ns: not significant; *p < 0.05; **p < 0.01; ***p < 0.001.

In order to assess the effects of acoustic over-exposure on the FVB/nJ mouse model, we first needed to determine the amount of noise exposure necessary to produce significant but reversible hearing threshold shifts. We chose 105 dB of 8–16 kHz single octave noise as our acoustic exposure for these experiments, varying the length of exposure and assessing temporary and permanent threshold shifts. We found that the FVB/nJ mouse strain is comparably susceptible to acoustic over-exposure relative to what has been reported for CBA/CaJ mice^18^,^19^,^20^,^21^,^22^. Significant threshold shifts of 30–40 dB were seen one day after the application of either 30 or 60 minutes of acoustic over-exposure (Fig. 1, cf. red and blue lines, p-value = 0.010, 0.008, 0.018, 0.010, 0.004 and 0.964, 0.070, 0.001, 0.001 and 0.001 for 30 minute or 60 minute treatment conditions respectively for 8–32 kHz frequencies; p-values for 14 days post-treatment vs pretreatment conditions were 0.003, 0.149, 0.247, 0.166, 0.153 for 30 minute treatment conditions, 2-way ANOVA with Tukey’s honest significant difference correction for both). Note that a 30 minute exposure of 105 dB has approximately 79% of the energy used for the 2 hour exposure of 100 dB used to induce reversible threshold shifts in CBA/CaJ mice. For 60 minute exposures at 105 dB, the exposure level was roughly one and a half times the conditions used for reversible threshold shifts for CBA/CaJ mice^21^,^23^ and resulted in an apparent threshold shift in FVB/nJ at all frequencies at day 14 post-treatment. This shift was not significant upon statistical evaluation, however (p-value = 0.728, 0.496, 0.976, 0.441 and 0.211 for 60 minute treatment conditions, 2-way ANOVA with Tukey’s honest significant difference correction). These two observations combined show comparable susceptibility for damage from acoustic over-exposure in FVB/nJ compared with CBA/CaJ of the same age. It is also interesting to note that while DPOAE threshold shifts were seen 1 day after acoustic over-exposure (p-value = 0.961, 0.314, 0.002, 0.074, 0.047, for 30 minute treatments and 1.000, 0.317, 0.038, 0.138, 0.102 for 60 minute treatments in pretest vs. day 1 post-treatment responses, two-way ANOVA with Tukey’s honest significant difference correction for both), DPOAE threshold shifts recovered completely in both treatments 14 days later (p-values for 30 minute and 60 minute noise treatments comparisons between pretest vs 14 post-treatment day responses were 0.541, 0.999, 0.467, 0.999, 0.676 and 0.017, 0.184, 0.551, 0.787, 0.230 respectively for 8–32 kHz frequency regions, two-way ANOVA with Tukey’s honest significant difference correction for both). The recovery of thresholds for DPOAE measurements at day 14 post-treatment combined with the incomplete recovery of ABR thresholds at day 14 post-treatment suggests that loss of hearing may not be directly dependent upon outer hair cell activity for FVB/nJ mice within the acoustic damage regimes tested. These results are consistent with previous reports of FVB/nJ noise sensitivity^13^.

**Figure 2:**
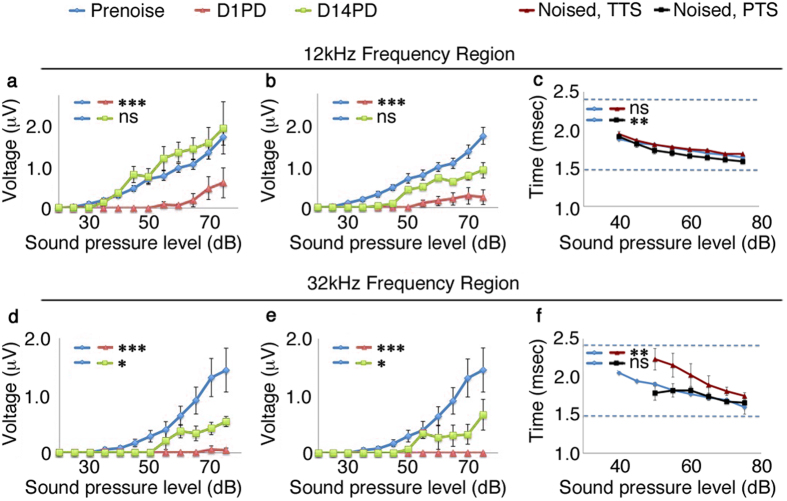
Temporary and permanent threshold shift conditions produce different effects on peak 1 amplitudes at 12 kHz and 32 kHz. (**a,b**) Voltage amplitude progressions for ABR peak 1 at 12 kHz or (**d,e**) 32 kHz prior to (cyan), 1 day after (red) or 14 days after (green) a 30 minute noise exposure (**a,d**) or a 60 minute noise exposure (**b,e**). Characterization of averaged ABR peak 1 latency values for (**c**) 12 kHz and (**f**) 32 kHz regions. Dashed lines in (**c,f**) indicate the collection windows for peak 1 assessment. Significant reductions in ABR amplitude are seen at 32 kHz 14 day after treatment. ns: not significant; *p < 0.05; **p < 0.01; ***p < 0.001.

Acoustic over-exposure may result in cochlear synaptopathy. One signature of cochlear synaptopathy is a loss of peak 1 ABR waveform amplitudes without loss of ABR threshold^5^,^21^. We measured peak 1 voltages of the ABR waveform for 12 kHz and 32 kHz frequency responses after both treatments, to determine if these amplitudes are also lowered in FVB/nJ mice after acoustic over-exposure as they are in CBA/CaJ mice (see^5^ for a similar example). We found that the 30 minute treatment induced an apparent reduction in amplitudes by S.E.M. at individual point assessments at 12 kHz on the next day. We employed the Wilcoxon rank sum test to assess the amplitude progression differences between the full data sets (Fig. 2a, p-value = 1.3e-09 using Wilcoxon rank sum test). These amplitudes recovered by 14 days after treatment (Fig. 2a, p-value = 0.0262, Wilcoxon rank sum test. Values are greater than the control condition.) The 60 minute treatment induced a next-day reduction in amplitudes at 12 kHz that also recovered (Fig. 2b, p-value = 3.135e-05 and 0.121 respectively for noise-induced shift and shift recovery at day 14 post-treatment, Wilcoxon rank sum test). At 32 kHz, FVB/nJ mice exhibited a complete loss of amplitude one day after both treatments, consistent with the loss of threshold (Fig. 2d,e, p-value = 7.756e-08 and 6.403e-05 for 30 minute and 60 minute treatments, Wilcoxon rank sum test). Only partial recovery of peak 1 voltages at 32 kHz was observed at 14 days after both treatments (Fig. 2d,e, 0.04137 and 0.0254 respectively, Wilcoxon rank sum test). Thus, a significant reduction in 32 kHz peak 1 amplitudes was observed in FVB/nJ mice after acoustic over-exposure without associated loss of DPOAE thresholds. It is interesting to note that latencies were slightly but significantly affected in the 12 kHz region for the 60 minute acoustic exposure condition compared to control (Fig. 2c, p-values = 0.46 and 0.002, 30 and 60 minute exposures respectively, Wilcoxon rank sum test). A significant change was also observed in the latency curves after 30 minutes acoustic exposure in the 32 kHz region (Fig. 2f, p-value = 0.0006 and 0.69, 30 minute and 60 minute exposures respectively, Wilcoxon rank sum test).

### Changes in cochlear inner hair cell synapses in FVB/nJ mice after acoustic over exposure

**Figure 3:**
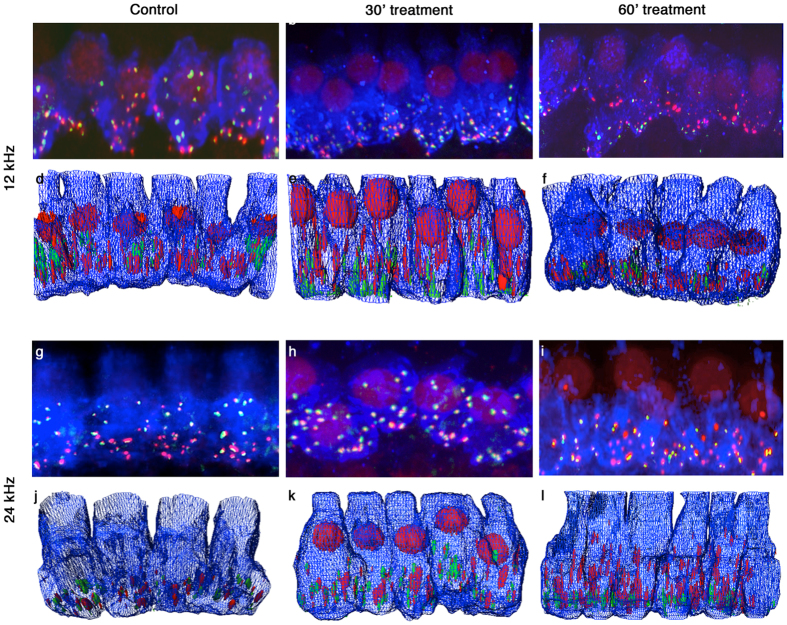
Effects of noise exposure on cochlear inner hair cells as visualized by confocal microscopy and 3D rendering. (**a–c**) Confocal imaging of Myosin VIIa (blue), Ctbp2 (red), and Gria2 receptor patches (green) in inner hair cells from the 12 kHz frequency region. Control tissue (**a**) is compared to tissue from (**b**) 30 minute and (**c**) 60 minute noise-exposed animals, 14 days after treatment. Deconvolution 3D rendering of optical sections recorded in (**a–c**) are shown in (**d–f**). (**g–l**) Same as (**a–f**), for 24 kHz inner hair cells.

We compared average numbers of synapses per inner hair cell in FVB/nJ mice exposed to either acoustic over-exposure or to untreated controls. Immunofluorescence and confocal microscopy were utilized to characterize the pre- and post-synaptic markers for inner hair cells mapped to specific frequencies within the cochlea (Fig. 3). Marker fluorescence blooms were subjected to deconvolution and reconstruction in order to correct for the light scattering effects of tissue within the whole mount optical sections. Reconstructions of IHCs from both frequencies are shown with each representative confocal stack. Unlike previous reports for experiments performed with CBA/CaJ mice, we did not find a strict 1:1 pairing of Ctbp2+ ribbon and Gria2+ receptor components in the untreated controls. The frequency of orphan or unpaired synaptic components increased after acoustic over-exposure, especially at 24 kHz after 60 minutes acoustic over-exposure (Fig. 3l). Others have previously described the presence of orphan ribbons in noise exposed animals^24^. We see similar effects in FVB/nJ. Myo7a staining showed no obvious change in cellular morphology 14 days after acoustic over-exposure (Fig. 3).

**Figure 4:**
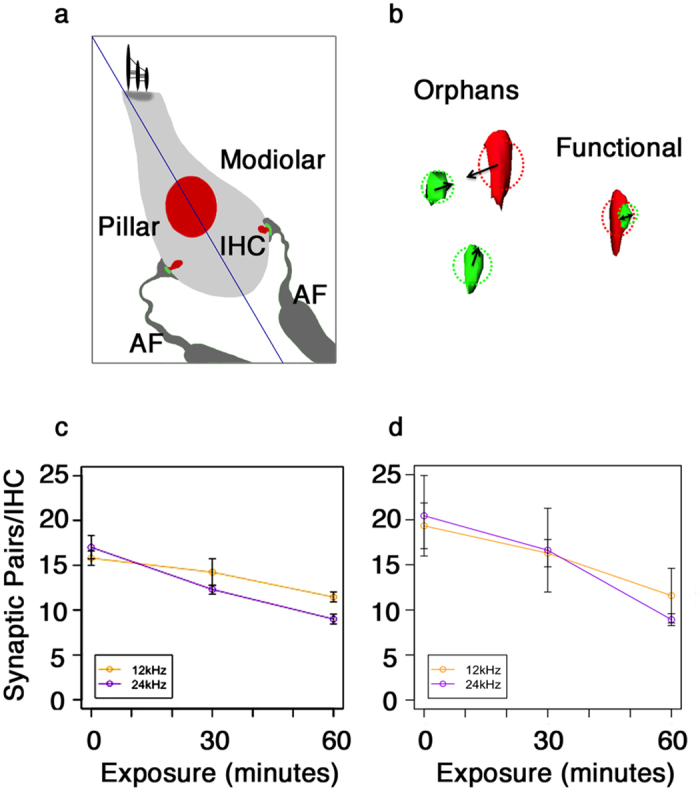
60 minutes noise exposure reduces the number of synaptic pairs in the 24 kHz frequency region. (**a**) Schematic representation of inner hair cell ribbon synapse and auditory nerve fiber position, with a bisecting axis running from the cuticular plate to the habenula-perforata. (**b**) Schema representing the assessment of synapse functionality derived from pre- and post-synaptic element proximity. (**c**) Visual quantification (human observer) of co-localized Ctbp2 and Gria2 domains. (**d**) Algorithmic identification of functional synapses by proximity. Error bars: s.e.m.

We wanted to quantitatively analyze synaptic components of inner hair cells after acoustic over-exposure. Automated identification of synaptic components could enable analysis of component volume and position as well as number. In order to automate the statistical analysis of these components, we employed a pair determination algorithm published previously^25^. This algorithm identifies paired synapses from reconstructions of independent staining for Ctbp2, the presynaptic ribbon marker, and Gria2, the post-synaptic receptor marker (Fig. 4b). Unpaired orphan Gria2 or Ctbp2 elements were not included in subsequent statistical treatments. Average numbers per hair cell are given in Table 1.

To test the algorithm, a human observer counted paired synaptic components on projected views of confocal stacks of inner hair cells for both 12 kHz (Fig. 4c, orange) and 24 kHz (Fig. 4c, purple) IHCs in control samples as well as ones from animals 14 days after each treatment. A statistically significant reduction of 37.5% in synaptic number was only observed for 24 kHz IHCs 14 days after 60 minutes of acoustic over-exposure (p = 0.0027, Bonferroni adjusted t-test). The computer algorithm was employed on the same confocal stacks (Fig. 4d). The constraints used to obtain average numbers through automated identification were set to produce similar results to those obtained from human identification. However, greater variation observed in automated identification negated statistical significance. Thus, there is a modest error associated with automated identification. We justify the use of automated identification, however, as we wish to further analyze the components of the synapses in terms of their positions and volumes. Without a method to specifically identify synaptic components in a thresholded reconstruction, background staining may be misclassified as synaptic components. We use proximity of pre- and post-synaptic components to each other as a way to automatically reduce the number of elements originating from background staining. Moreover, we are also interested in comparing changes of presynaptic component volumes as a function of post-synaptic volumes. This analysis require pairing of the components.

**Figure 5:**
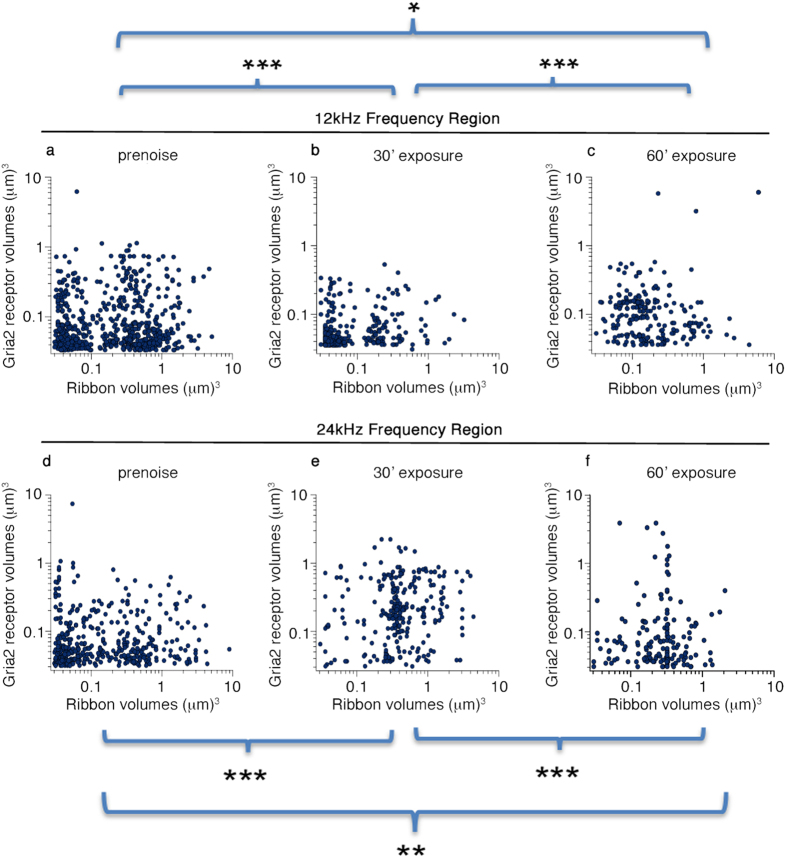
Synaptic component volumes are dynamically regulated in response to noise exposure. (**a–c**) Analysis of deconvoluted volumes of paired synapse elements of 12 kHz inner hair cells for (**a**) pre-noise condition, (**b**) 30 minute, and (**c**) 60 minute noise exposure. (**d–f**) Same analysis for 24 kHz inner hair cell synaptic pairs. Statistical comparisons are shown by bars with indicated levels of significance. The volumes of 12 kHz IHC synaptic components change significantly after noise but the presence of both small and medium sized ribbons is preserved. Small ribbons are absent in 24 kHz IHCs 14 days after noise exposure.

We analyzed the volume information for both synaptic components to identify dynamic changes after acoustic over-exposure. For 12 kHz IHCs, when Ctbp2 volumes are plotted against Gria2 volumes, the distributions may not appear greatly affected, although statistically they are very different (Fig. 5a–c, p-value = 2.2e-16|1.024e-15 and 0.0137|0.143 for 0 versus 30, and 0 versus 60 minute treatment conditions for Ctbp2|Gria2, Wilcoxon rank sum test). These changes in volume do not appear to be associated with synaptic losses (Fig. 4).

Synaptic component dynamics are even more pronounced at the 24 kHz region. Recall that while both treatment conditions reduce peak 1 measures (Fig. 2a,b,d,e), in the 24 kHz region only 60 minutes of acoustic over-exposure significantly reduces synaptic number (Fig. 4). After the 30 minute acoustic over-exposure, the prevalence of both small and large ribbons is reduced, and relatively more medium-sized ribbons are observed (Fig. 5, cf. d,e, p = 6.209e-08, for pairwise comparison, Wilcoxon rank sum test). These data indicate that ribbon volume in the cochlea is dynamic, because this is the condition in which synaptic number is preserved. Gria2+ patch volumes also appear to be shifted larger (Fig. 5, cf. d,e, 1.236e-07, for pairwise comparison, Wilcoxon rank sum test). The 60 minute acoustic over-exposure affects ribbon size in 24 kHz IHCs similarly to the shorter treatment (Fig. 5, cf. d,e, p-value = 1.384e-11 for pairwise comparison, Wilcoxon rank sum test). In addition, large Gria2 receptor patches are now depleted (p-value = 4.6e-05 for pairwise comparison, Wilcoxon rank sum test). This loss of synapses containing large receptor patches is concurrent with the previously determined reduction in synaptic count per hair cell (Fig. 4).

We wanted to determine if the synaptic components that displayed the most changes were located on the pillar or modiolar side of the inner hair cell. To this end, the positions of paired synaptic components within stacks were rotated and projected onto the habenular-cuticular and modiolar-pillar position axes of inner hair cell optical sections (Fig. 4a). Alignment of z-projections of paired synapses was performed by fitting each optical stack to a single Gaussian distribution model via maximum-likelihood estimation. The centroid for the paired ribbon synapse population of each optical stack was used for alignment. The centroids of all synapse populations were adjusted to identical x,y-coordinates for all optical stacks for construction of the final ensemble.

**Figure 6:**
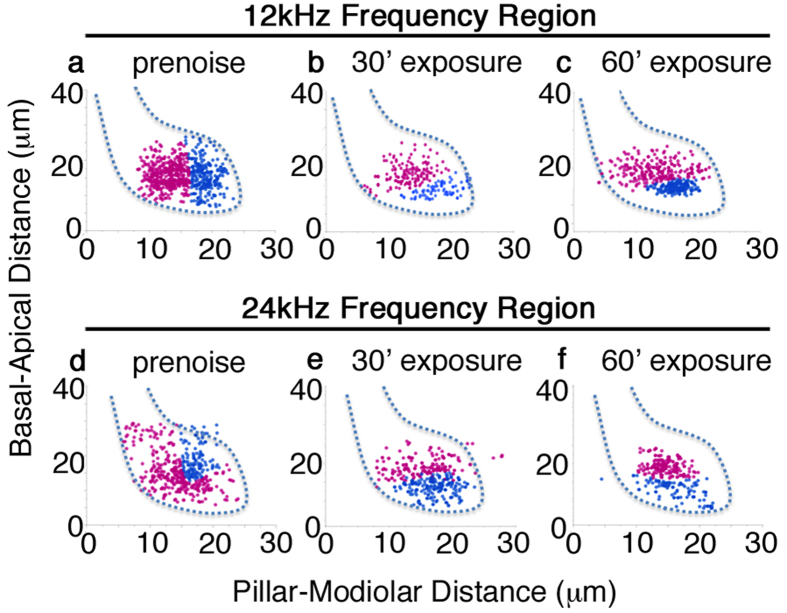
Synapse position is affected by noise exposure. Projected paired synapses referenced to cell membrane shape in 12 kHz frequency region for (**a**) pre-noise; (**b**) 30 minute and (**c**) 60 minute noise exposure. Color choice of two populations is arbitrary. (**d–f**) Same analysis for 24 kHz inner hair cells. Synaptic positioning is more compact after noise exposure in 24 kHz IHCs.

We wondered if synaptic clusters would display segregation along a modiolar and pillar axis^24^,^25^,^26^ as indicated in Fig. 4a. Figure 6 shows the positions of synapses for 12 kHz (Fig. 6a–c) and 24 kHz IHCs (Fig. 6d–f) for controls and both acoustic over-exposures. In control samples for both frequencies, there is a rough axis of symmetry dividing the two populations of synapses into pillar and modiolar groups when inner hair cells are partitioned from cuticular-plate to habenular-pole (Fig. 6a,d)^27^. The 24 kHz clustering separation is less consistent. For both 12 and 24 kHz IHCs, 30 minutes of acoustic over-exposure appears to drive a rotation of this axis at 14 days post-treatment. The 60 minute treatment shows a similar axis rotation in both 12 and 24 kHz IHCs 14 days post-treatment. These data indicate that acoustic over-exposure impacts precise synaptic positioning. Although the clustering algorithm is not helpful for identifying areas of synaptic loss, by comparing 24 kHz IHCs after 60 minutes of acoustic over-exposure to the control it is evident that the remaining synapses are predominately towards the habenular pole and centralized (Fig. 6f, cf. to d).

**Figure 7:**
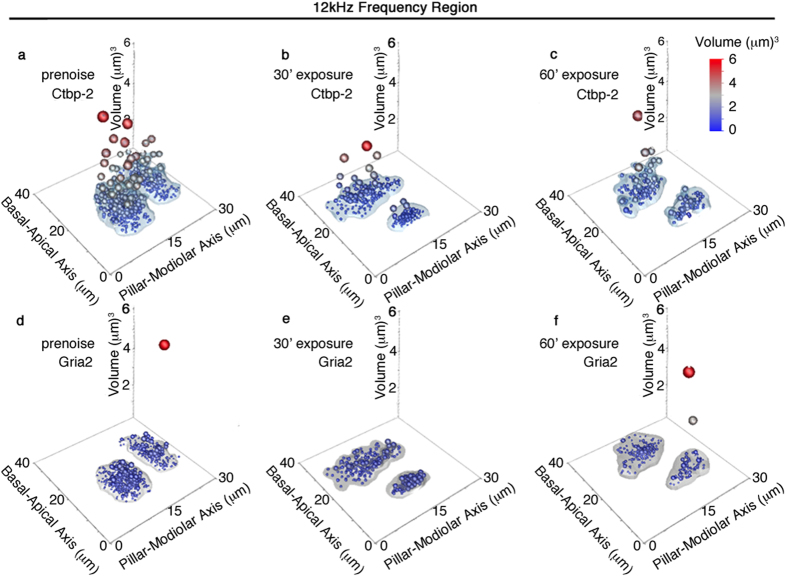
Spatial localization of large synaptic components after noise exposure in the 12 kHz frequency region. (**a–c**) Deconvoluted volumes for Ctbp2 fluorescence are shown with 0.9 quantile assessment for (**a**) prenoise, 30 minute (**b**), and 60 minute (**c**) noise exposure. Large ribbons are observed on the cuticular (basal) region of the IHCs in all three conditions. (**d–f**) 0.9 quantile assessment for corresponding deconvoluted Gria2 fluorescence volumes. Points are sized in accordance with synaptic volume to reveal the spatial distribution of outlier synapses.

It is interesting to see which ribbons are preferentially affected by acoustic over-exposure. A clustering analysis was performed to examine the effects of treatment on outlier (larger) components in both sub-populations of ribbons (Fig. 7a–c) and Gria2 patches (Fig. 7d–f) at 12 kHz. Large, volumetric outliers are clearly observable by the lack of continuity associated with the blue and white shaded regions indicating presence within the 0.9 quantile for control IHCs. Prior to acoustic over-exposure, it appears that large ribbons are positioned towards the cuticular plate, often towards the pillar side of 12 kHz IHCs (Fig. 7a). After acoustic over-exposure, large outlier ribbons throughout the IHC become less prevalent by day 14 post-exposure although those remaining maintain their position. Gria2 patches are not significantly inhomogenous for all acoustic over-exposure conditions tested (Fig. 7d–f).

**Figure 8:**
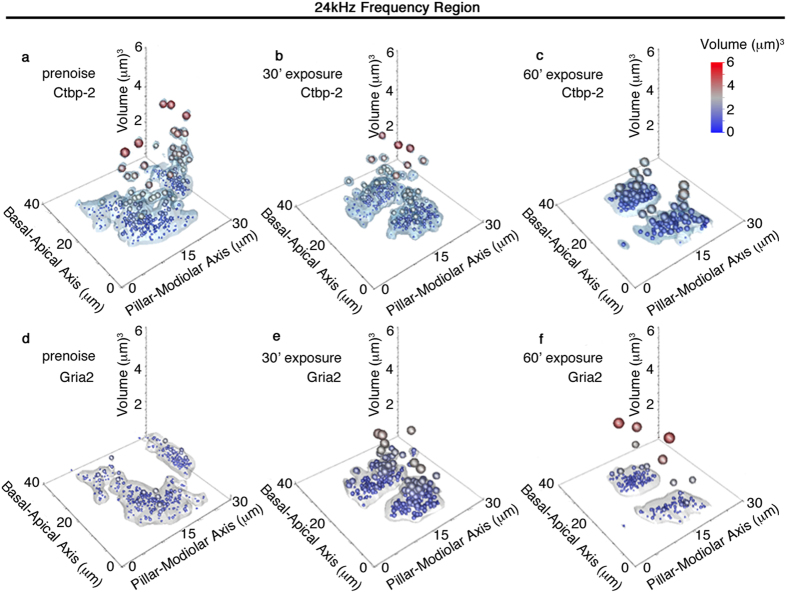
Spatial localization of large synaptic components after noise exposure in the 24 kHz frequency region. (**a–c**) Deconvoluted volumes for Ctbp2 fluorescence are shown with 0.9 quantile assessment for (**a**) prenoise, 30 minute (**b**), and 60 minute (**c**) noise exposure. Large ribbons are cuticular (basal) and modiolar in the prenoise and 30 minute exposure condition; however, they are distributed throughout the IHC after 60 minutes of noise. (**d–f**) 0.9 quantile assessment for corresponding deconvoluted Gria2 fluorescence volumes. Larger Gria2 + components are distributed cuticularly (basal) after 30 minutes of noise exposure. Points are sized in accordance with synaptic volume to reveal the spatial distribution of outlier synapses.

Large ribbon prevalence is particularly dynamic in 24 kHz IHCs (Fig. 8b,c and cf. with 8a) in this positional, volumetric outlier analysis. Prior to noise exposure, it appears that large ribbons are positioned more often towards the cuticular plate, but distributed between the pillar and modiolar sides in 24 kHz IHCs (Fig. 8a). Untreated Gria2 patches at 24 kHz are not significantly different from 12 kHz (Figs 8d, cf. with 7d). 30 minutes of acoustic over-exposure reduces the prevalence of large ribbons, but increases the prevalence of larger Gria2 patches throughout the inner hair cell 14 days later (Fig. 8b,e). 60 minutes of acoustic over-exposure results in a smaller population of more homogenous-sized ribbons (Fig. 8c). The few observed outliers are now found towards the habenular pole (Fig. 8c). Gria2 patches are more similar to the untreated sample with the addition of significant outliers (Fig. 8f).

**Figure 9:**
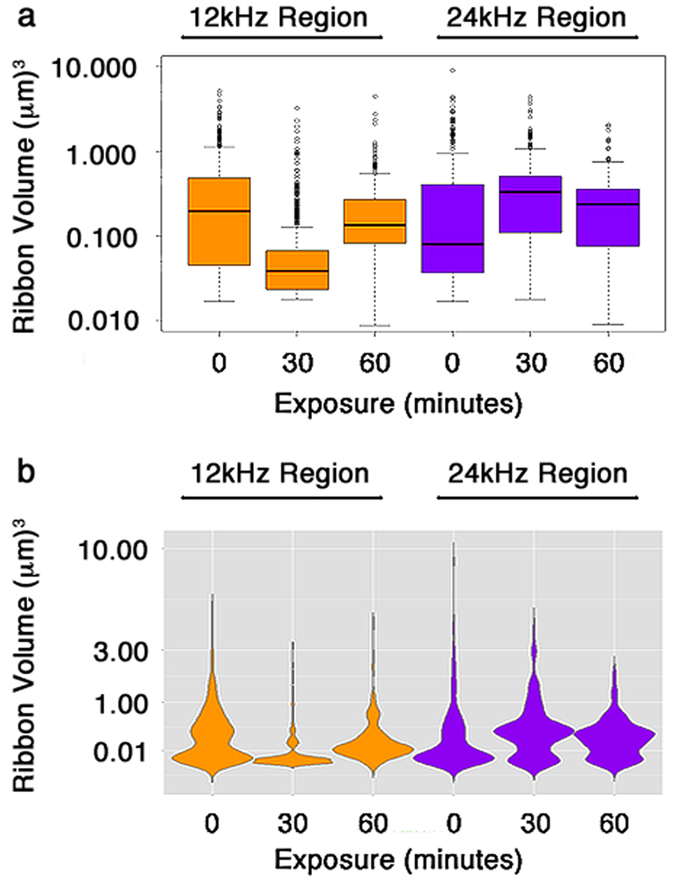
Variance analysis better captures synapse conditioning dynamics over means. Means (**a**) and smoothed kernel density histogramming (**b**) analysis of ribbon sizes in response to 12 kHz prenoise, 30 minute, and 60 minute noise exposure treatments in the 12 kHz and 24 kHz frequency regions of the cochlea. A clearer picture of the variants as well as the change in population moments is shown in (**b**) for 12 kHz and 24 kHz frequency regions. The mean shift is actually in the opposite direction as the outlier populations for the 24 kHz region whereas the 12 kHz region shows similar dynamics.

We quantified the effects of acoustic over-exposure on outlier ribbons within 12 kHz and 24 kHz IHCs (Fig. 9). An analysis of the means and the overall variance offers a way to determine the resizing dynamics of ribbons in response to noise for IHCs of each frequency. We found that mean volumes for high and low frequency IHCs are dynamically shifted during noise exposure. For 12 kHz IHCs (Fig. 9a, orange boxes), mean ribbon sizes initially decrease with a contraction in outlier sizes after a 30 minute treatment. This is followed by a further increase in mean size with further contraction of outliers after a 60 minute treatment. For 24 kHz IHCs (Fig. 9a, purple boxes), the mean volume increases after both treatments. We visualized the variances in ribbon size for both 12 kHz and 24 kHz IHCs via violin plots of Gaussian kernel smoothed density functions (Fig. 9b). A profound loss of variance is observed after both treatments for 24 kHz IHCs, where both large and small synaptic ribbons revert to the mean. These data are consistent with the interpretation that noise exposure causes complex dynamics in both volume and position of synaptic components, which may correlate with hearing dysfunction^5^,^28^,^29^.

## Discussion

Acoustic exposure can permanently alter the ability to hear in background noise, a pathology called auditory neuropathy in humans. In animals, acoustic exposure can affect the high-frequency suprathreshold responses in the ABR, which may reflect compromised signaling ability. These changes can correlate with the loss of inner hair cell synapses, called cochlear synaptopathy, but they also occur in its absence. Here we correlate changes in synaptic component volumes with the presence of reduced suprathreshold responses in FVB/nJ mice, which occur in the absence of synaptopathy. Our use of deconvolution enables a more precise estimation of synaptic component volumes, revealing a previously unseen complex dynamic. These studies also set a baseline for analyzing noise damage in a congenic mouse model that should be amenable to later genetic analyses.

Cochlear synaptopathy in humans remains an under-reported problem which continues to impact the lives of people of all ages. Noise induced cochlear synaptopathy is a specific kind of auditory injury which deeply impacts the lives of those affected, with particular negative quality of life consequences in older workers and military veterans. In mouse models of noise induced hearing damage, specifically CBA/CaJ, it has been shown that inner hair cell synapses can be compromised^5^,^30^. Moreover, mechanotransduction elements may also be damaged during noise exposures^31^. This may manifest in a hearing loss pathology where damage has occurred either before or in concert with the loss of synapses observed in cochlear synaptopathy. Indeed there are cases consistent with this observation where hearing threshold shifts are seen which do not show a direct relation to outer hair cell losses^6^. Predictively, the observed effect assayed by ABR and DPOAE measurements would be a reversible outer hair cell associated DPOAE threshold shift correlated with a permanent ABR threshold shift.

From the threshold shift data in Fig. 1a,c, we see that 30 minutes noise exposure causes a significant threshold shift in both ABR and DPOAE that recovers in 14 days. This is the normal threshold shift dynamic seen in cochlear synaptopathy acoustic treatment conditions in young CBA/CaJ mice^28^. For the longer exposure time shown in Fig. 1b,d, a reversible DPOAE shift is concomitant with an apparent ABR threshold shift; however, the latter does not reach statistical significance. Both the shorter and longer noise exposures give rise to permanent reductions in peak 1 voltage amplitudes in the higher frequency (Fig. 2d,e), which is also consistent with the effect of noise exposure in CBA/CaJ^5^,^6^,^30^. That FVB/nJ mice have a fairly similar responses to CBA/CaJ lends credence to their use as a model for noise damage genetics in mice.

Acoustic overexposures in CBA/CaJ mice have been consistently shown to induce differential effects on synapse integrity^1^,^24^ depending upon the level of noise. Permanent and temporary threshold shifts resulting from noise damage in these exposure conditions have been strongly correlated with synaptic losses, resulting in cochlear synaptopathy^1^. The 30 minute noise exposure we used is below the sound exposure level necessary to induce cochlear synaptopathy in CBA/CaJ. FVB/nJ do not have significantly reduced visual counts of inner hair cell paired synapses in either frequency region of the cochlea for this exposure (Figs 3 and 4), underlining the similarity between the mouse strains. For the 60 minute noise exposure, we find that synapses are clearly lost in the mid-range frequency lamina of the cochlea. This middle frequency region has been shown to be the most susceptible to noise damage in CBA/CaJ and we see the same effect in FVB/nJ (Fig. 3i,l). The molecular basis for synapse preservation could be analyzed with these noise levels, and would be an interesting problem to examine using genetic tools.

The synaptic component sizes and positions analyzed in this paper were obtained from confocal stacks after deconvolution using the iterative maximum likelihood estimation package included with the AMIRA FEI software suite. Deconvolution is important because fluorescence microscopy has artifacts: significant numbers of fluorescence photons are scattered by tissue outside of the image plane. This problem is dependent on wavelength and penetration depth. It is also tissue dependent, due to the variable scattering cross-section of support and membrane proteins, a particular problem for whole mount samples. The total photon scattering convolves the acquired data with a point spread function to make objects appear larger. Unfortunately, convolution is non-linear, in that smaller bright objects appear similar in volume in raw data stacks to medium sized but dimmer objects at any particular thresholding level. The data must be deconvolved from the point spread function plane-by-plane due to the changing nature of the stack depth and scattering centers. In this way we gained more accurate positional and volumetric data for analysis.

Deconvolved confocal imaging provides the ability to assay fine structure dynamics of most of the ribbon synapse populations. Through averaging in deconvolved optical stack reconstructions, we are able to assay small, medium, and large ribbon populations independently, something not possible from serial electron microscopy, pure confocal, or super-resolution imaging experiments. We pair this with algorithmic synapse identification based on pre- and post-synaptic element proximity. Thus, we are able to do an analysis of fine structure in each ribbon volume distribution. Ribbon volumes in particular are interesting because auditory nerve fiber spontaneous rates may be related to ribbon synapse size^32^. Indeed Furman *et al*.^24^ have shown that mean ribbon sizes are modified by noise exposures to different levels in different frequency regions in guinea pig cochlea, although they did not assess fine structures.

By plotting synapse anatomical domain volumes against each other (Fig. 5a–f) we show that the 12 kHz inner hair cells possess a population of small ribbons that persist following noise exposure. This is not true in the case of the 24 kHz inner hair cells, the small ribbon population is severely depleted after both 30 minute and 60 minute noise exposures. Because there is no significant change in synapses number at 24 kHz after 30 minute noise exposure (Fig. 4c), this suggests that ribbons are resized in response to mild noise insult. Strikingly, this also correlates with suprathreshold amplitude losses in the 32 kHz region (Fig. 2d,e). The drawback to this analysis is that there is no direct connection to suprathreshold amplitudes because we are examining only anatomical correlates. Further work will be needed to fully characterize this effect and to determine if it is observable in other animal models such as guinea pig or CBA/CaJ.

The changes we observe in resizing dynamics are also shown to occur in concert with changes in position of synapses after noise exposure. We utilized Bayesian cluster analysis to determine the amount of positioning fidelity inherent to synapses arranged about the cuticular-habenular axis before and after acoustic over-exposures. In the control cases for both 12 kHz and 24 kHz, population clusters are observed to segregate roughly parallel to the cell body axis. Following noise exposure, positioning is altered for both frequency regions examined. These changes do not correlate with ABR peak 1 amplitudes reductions. The noise exposure seems to disrupt synapse position along the soma of the inner hair cells, however we are unable to determine if modiolar local synapses are affected differently compared to pillar synapses at this time.

The positional dynamics of resizing of the anatomical correlates are interesting to consider. The 12 kHz (Fig. 7) and 24 kHz (Fig. 8) regions are shown to have different effects in response to noise. In the 12 kHz case, outlier (large) ribbons are predominately cuticular. Although resizing is observed, large ribbons persist in that location. There are comparatively few changes seen in the Gria2 volumes at 12 kHz. For the 24 kHz frequency region, large ribbons were shown to be predominantly modiolar in aspect. When noise was applied for 30 minutes, fewer large ribbons are seen, but their position remains modiolar. For 60 minute noise exposures, large ribbons are distributed more throughout the inner hair cell. The Gria2 patches on the other hand, increase substantially after 30 minutes of noise exposure and they are located towards the modiolar side. These large patches are lost during the 60 minute exposure.

Mean ribbon sizes offer an additional metric for comparison of FVB/nJ to CBA/CaJ. Comparison of means in FVB/nJ (Fig. 9a) with mean ribbon volume data for guinea pig shows a high degree of similarity^24^. Our analysis (Fig. 9b) also shows that the low and mid-frequency regions react differently to noise exposure. Most evident is the fact that larger components are not as drastically affected in the low frequency region. In contrast, at 24 kHz, ribbons sizes seem to collapse to a central value in response to noise over exposures. This is interesting because the individual moments of the ribbon distributions seem to converge. Perhaps there is an optimal size for ribbons to resist damaging noise levels.

## Materials and Methods

### Animals and groups

Postnatal day 1 congenic FVB/nJ mice were purchased from Jackson Laboratories, raised in our facility and used for characterization of the effects of acoustic overexposure. All mice were randomized for treatment condition. Both males and female mice were used for all experiments. The numbers of mice used per condition were 20 mice for controls, 5 mice for 30 minute noise treatment condition, and 3 mice for the 60 minute treatment condition. They were pretested for auditory function and then noise exposed at P60. Animals were kept for 14 days post treatment, re-tested, and sacrificed. All experiments were performed in accordance with the Department of Health and Human Services and were approved by the University Committee on Animal Resources at the University of Rochester Medical Center.

### Acoustic over-exposures

Noise exposure conditions were a single octave of 8–16 kHz band Gaussian noise at 105 dB SPL for either 30 or 60 minutes. Mice were placed into individual triangular mesh wire cages, 12 cm × 5 cm × 5 cm, in an asymmetric plywood box. The ceiling of this enclosure was modified to connect with a mounted JBL2250HJ compression speaker and a JBL2382A bi-radial horn. The entire system was enclosed within a soundproof booth and was driven using a TDT RX6 multifunction processor and dedicated attenuator. This was controlled using TDT TPvdsEx sound processing software.

### Physiological tests

Auditory testing was conducted using a Smart EP Universal Smart Box (Intelligent Hearing Systems). P60 FVB/nJ mice were tested for hearing thresholds via ABR and DPOAE responses. Mice (respective by acoustic treatment condition-Control, 30 minute, 60 minutes) were anesthetized by intraperitoneal injection using a mixture of ketamine (80 mg/kg) in a sterile acepromazine/saline mixture (3 mg/kg). A 10B+ high-frequency transducer probe was placed within the external auditory canal. Auditory brainstem response stimuli were either 5 ms full tone spectrum clicks or 5 ms single tone pips presented at 8, 12, 16, 24 and 32 kHz. Each set of frequency and amplitude measurements were averaged for 512 scans with electrical responses being reported via a 3-electrode system. The three needle electrodes (Grass) were inserted at the vertex (ground) and on each side beneath the pinna. ABR thresholds were determined by the last trace corresponding to a correct latency and waveform by visual identification for a particular stimulus. The individual scoring thresholds was blinded to condition and time point. Threshold response determinations were performed by collection of ABR response using 75–15 dB SPL single tones or a click stimulus. ABR amplitude progressions were taken by reporting the ABR peak 1 voltages for these measurements. Amplitudes and latencies of ABR peak 1 were identified using IHS Software analytical tools. Peak 1 was identified as the first peak in the waveform, provided it occurred within 2.4 msec of the stimulus presentation.

Distortion product otoacoustic emissions (DPOAE) measurements were determined by measuring amplitudes of the distortion product response. Evoked emissions were recorded at frequencies for paired tones of f1 and f2 with f1/f2 = 1.2 and f1 level = f2 + 10 dB. Averages of 32 sweeps were used in reporting 5 dB f1 and f2 steps with f1 starting with 20 dB and ending with 75 dB. 3 dB was used as the DPOAE threshold. The value at 3 dB was calculated from a linear interpolation of the surrounding points.

### Antibodies

Primary antibody were used as follows: rabbit anti-Myosin7a (1:200; Santa Cruz Biotechnology), mouse anti-Ctbp2 (1:200; BD Transduction Laboratories), and mouse anti-Gria2 (aka GluR2/GluA2; 1:2000; Millipore). Secondary antibodies were all purchased from Jackson Laboratories and were used as follows: goat anti-mouse 488 (1:500), goat anti-mouse 594 (1:500), donkey anti-rabbit 647 (1:200).

### Histologic preparation, confocal imaging, synaptic counts and reconstruction

Cochleae were removed via dissection and had their stapes removed. The round window membranes were opened and cleared of tissue and a small hole was placed at the tip of the cochlea to permit fluid exchange. Cleared organs were fixed in 4% paraformaldehyde for overnight at 4 **°**C. Decalcification was carried out in EDTA solution (0.1 M) at 4 **°**C on a rocking platform for 5 days. Synapse analysis were performed on whole mount preparations by microdissection as described elsewhere^25^. Frequency mapping was carried out prior to immunostaining using the ImageJ plugin developed by the Massey Eye-Ear Institute’s Eaton-Peabody Laboratories (available at http://rsbweb.nih.gov/ij/).

Confocal imaging was performed using an Olympus FV 1000 laser scanning confocal microscope. Visualization and projections were done using FIJI and ImageJ 64. Visual quantification of pairing in synaptic structures was done by counting unpaired Ctbp2+ ribbons and Gria2+ receptor patches in each optical stack. Counting was performed using the ImageJ 64 “cell counter” plugin and up to 2 stacks were counted per organ.

The synaptic component sizes and positions analyzed in this paper were obtained from confocal stacks after deconvolution using the iterative maximum likelihood estimation package included with the AMIRA FEI software suite. Algorithmic pair determinations on high-resolution reconstructions were performed as described previously^25^. To identify synaptic clusters, the resulting populations were sorted by an expectation-maximization (EM) algorithm utilizing Bayesian identification of clusters^33^,^34^. Briefly, EM seeks to determine the maximum likelihood parameters of a data set, in this case set to a Gaussian statistics model. The likelihood of all data points as belonging to a model distribution is computed and cluster assignments are determined based upon a Bayes information criterion scoring which incorporates a penalty for over-assignment of clusters. For the analysis we have employed, we assume that the modiolar-pillar distribution model is correct and therefore yields two independent populations of ribbon synapses.

## Additional Information

**How to cite this article**: Paquette, S. T. *et al*. Noise exposure modulates cochlear inner hair cell ribbon volumes, correlating with changes in auditory measures in the FVB/nJ mouse. *Sci. Rep*. **6**, 25056; doi: 10.1038/srep25056 (2016).

## Figures and Tables

**Table 1:** Paired and orphan ribbons per inner hair cells.

Condition	Frequency Region	Organs analyzed	Total ribbons/IHC	Paired ribbons/IHC	Orphan ribbons/IHC
Control	12 kHz	3	17.3 ± 0.6	15.8 ± 0.8	1.5 ± 0.2
	24 kHz	3	18.4 ± 1.2	17 ± 1.3	1.4 ± 0.2
30 min	12 kHz	4	16.1 ± 1.0	14.2 ± 1.5	1.9 ± 0.5
	24 kHz	3	15.3 ± 0.3	12.3 ± 0.5	2.9 ± 0.5
60 min	12 kHz	4	14.4 ± 0.7	11 ± 0.6	3.4 ± 0.5
	24 kHz	4	13.5 ± 0.8	9 ± 0.5	4.5 ± 1.2

Summary of tissue analyzed in this report, with the visual determination of ribbon number per inner hair cell that are either paired with Gria2 receptor patches or not (“orphan”).
